# Ovarian Cancer Spheroid Cells with Stem Cell-Like Properties Contribute to Tumor Generation, Metastasis and Chemotherapy Resistance through Hypoxia-Resistant Metabolism

**DOI:** 10.1371/journal.pone.0084941

**Published:** 2014-01-07

**Authors:** Jianqun Liao, Feng Qian, Nana Tchabo, Paulette Mhawech-Fauceglia, Amy Beck, Zikun Qian, Xinhui Wang, Wendy J. Huss, Shashikant B. Lele, Carl D. Morrison, Kunle Odunsi

**Affiliations:** 1 Departments of Gynecologic Oncology, Roswell Park Cancer Institute, Buffalo, New York, United States of America; 2 Immunology; Roswell Park Cancer Institute, Buffalo, New York, United States of America; 3 Center for Immunotherapy; Roswell Park Cancer Institute, Buffalo, New York, United States of America; 4 Surgical Oncology, Roswell Park Cancer Institute, Buffalo, New York, United States of America; 5 Urology Oncology, Roswell Park Cancer Institute, Buffalo, New York, United States of America; 6 Pharmacology & Therapeutics, Roswell Park Cancer Institute, Buffalo, New York, United States of America; 7 Pathology, Roswell Park Cancer Institute, Buffalo, New York, United States of America; 8 Surgical Oncology, Massachusetts General Hospital, Harvard Medical School, Boston, United States of America; 9 Atlantic Health System Morristown/Overlook Medical Centers, Women’s Cancer Center, Morristown and Summit, New Jersey, United States of America; Cedars-Sinai Medical Center, United States of America

## Abstract

Cells with sphere forming capacity, spheroid cells, are present in the malignant ascites of patients with epithelial ovarian cancer (EOC) and represent a significant impediment to efficacious treatment due to their putative role in progression, metastasis and chemotherapy resistance. The exact mechanisms that underlie EOC metastasis and drug resistance are not clear. Understanding the biology of sphere forming cells may contribute to the identification of novel therapeutic opportunities for metastatic EOC. Here we generated spheroid cells from human ovarian cancer cell lines and primary ovarian cancer. Xenoengraftment of as few as 2000 dissociated spheroid cells into immune-deficient mice allowed full recapitulation of the original tumor, whereas >10^5^ parent tumor cells remained non-tumorigenic. The spheroid cells were found to be enriched for cells with cancer stem cell-like characteristics such as upregulation of stem cell genes, self-renewal, high proliferative and differentiation potential, and high aldehyde dehydrogenase (ALDH) activity. Furthermore, spheroid cells were more aggressive in growth, migration, invasion, scratch recovery, clonogenic survival, anchorage-independent growth, and more resistant to chemotherapy *in vitro*. ^13^C-glucose metabolic studies revealed that spheroid cells route glucose predominantly to anaerobic glycolysis and pentose cycle to the detriment of re-routing glucose for anabolic purposes. These metabolic properties of sphere forming cells appear to confer increased resistance to apoptosis and contribute to more aggressive tumor growth. Collectively, we demonstrated that spheroid cells with cancer stem cell-like characteristics contributed to tumor generation, progression and chemotherapy resistance. This study provides insight into the relationship between tumor dissemination and metabolic attributes of human cancer stem cells and has clinical implications for cancer therapy.

## Introduction

Epithelial ovarian cancer (EOC) is the leading cause of death from gynecologic malignancies. There are more than 23,000 cases annually in the United States, and 14,000 women can be expected to die from the disease [1]. Despite modest improvements in response rates, progression-free and median survival using adjuvant platinum and taxane chemotherapy following cytoreductive surgery, overall survival rates for patients with advanced EOC and ovarian-like malignancies (primary peritoneal) remain disappointing [2]. This has been attributed to several reasons. First, in contrast to most other solid tumors, more than 75% of EOC patients present with advanced stage disease (FIGO III or IV). Secondly, although most patients initially respond to platinum and paclitaxel chemotherapy including complete responses, the relapse rate is approximately 85%. Within 2 years of cytoreductive surgery and systemic chemotherapy, tumors usually recur and once relapse occurs, curative therapy is difficult. Therefore, it is imperative to understand the mechanism(s) of EOC metastasis and chemotherapy resistance in order to improve clinical outcomes in this disease.

The primary mode of distant metastasis in EOC involves the shedding of cells from the primary tumor, into the abdominal cavity, followed by implantation on the mesothelial lining of the peritoneum [3], [4]. Currently, there are data to demonstrate that “sphere forming cells” or “spheroids” are commonly found in ascites and are capable of tumorigenesis *in vivo*, have a reduced response to chemotherapeutic drugs *in vitro,* and may play an important role in metastatic disease [5]–[9]. Because metabolic changes may confer an advantage on the ability of cancer cells to survive, proliferate, and invade [10]–[12], we hypothesized that sphere forming cells are likely to exhibit metabolic attributes that promote their ability to survive and metastasize. In present study, we generated spheroid cells from EOC cell lines and from patients with primary ovarian cancer. Our *in vivo* and *in vitro* biologic studies suggested that these sphere forming cells are enriched in cancer stem-like cells (CSCL) that critically contribute to ovarian cancer tumorigenesis, metastasis and chemotherapy resistance. We then utilized isotope-based dynamic metabolic profiling [13], [14], to simultaneously assess the substrate flux within and among major metabolic pathways of macromolecule synthesis and energy production under various physiologic conditions. We found that spheroid cells increase anaerobic glycolysis and pentose cycle and decrease re-routing of glucose for anabolic purposes. This study provides insights into the relationship between tumor dissemination and metabolic attributes of ovarian CSCL cells, and has clinical implications for cancer therapy.

## Materials and Methods

### Isolation of Tumor Cells from Human Ovarian Cancer

Tumor specimens and ascitic fluid were obtained from patients undergoing tumor debulking surgery for epithelial ovarian cancer (EOC) at Roswell Park Cancer Institute (RPCI), Buffalo, NY. All specimens were collected under an approved protocol CIC 02–15 from the Institutional Review Board at RPCI, and informed written consent was obtained from each patient. Tumor cells from ascites were obtained from centrifuged cell pellets of ascitic fluid. The pellets were washed twice in PBS, placed on Ficoll-Hypaque density gradients and centrifuged again to harvest tumor cells. To obtain tumor cells from solid tumor tissue, tumor specimens were finely minced in cell culture medium and single cell suspensions were washed twice in PBS followed by Ficoll-Hypaque purification.

### Cell Culture

Primary EOC cell lines were established from solid tumor and ascites by culturing cells in 13 different conditions [15], [16] from 30 EOC patients over a period of 2 years. Spheroid cells were generated from new EOC cell lines and from an established ovarian cancer cell line, OV2774, which were obtained from Sloan Kettering Institute, New York, NY (courtesy of Lloyd J. Old, Ludwig Institute for Cancer Research, NY), by the method as described [17] with modifications by resuspending 8×10^4^ cells with serum-free DMEM/F12 supplemented with 10 ng/mL human recombinant epidermal growth factor (EGF; Invitrogen), 10 ng/mL basic fibroblast growth factor (bFGF; Invitrogen), and N2 supplement-A (Stemcell Technologies Inc) in Ultra Low Attachment 6-well plates (Corning) and subsequent organization into spheres.

### *In vivo* Xenograft Experiments

All animal studies adhered to protocols approved by the Institutional Animal Care and Use Committee of RPCI. Dissociated spheroid or parent tumor cells were counted, resuspended in 50 µL 1∶1 RPMI/Matrigel (BD Biosciences), and injected subcutaneous (s.c.) into the right legs of 3- to 4-wk-old female SCID mice (C.B-igh-1blcrTac-Prkdcscid/Ros) provided by RPCI Animal Facility (originated from Taconic Farms, Hudson, NY). Engrafted mice were inspected biweekly for tumor appearance by visual observation and palpation, and tumor latencies were determined. Mice were sacrificed by cervical dislocation at a tumor diameter of 1 cm or at 6 months post-transplantation. Xenograft tumors were resected, fixed in 10% neutral, buffered formalin, and embedded in paraffin for sectioning (5 µm) on a rotary microtome, followed by slide mounting, H&E staining, and histologic assessment by a pathologist for tumor type, grade, and stage. To determine xenograft recapitulation of the parental tumor phenotype, the same process was performed on human tumors. To evaluate formation of ovarian tumors in their native environment, SCID mice were injected intraperitoneal (i.p.) with various amounts of spheroid-derived cells or their parent tumors, monitored biweekly for weight change and ascites formation, and euthanized upon excessive abdominal distention or palpable tumor growth.

### Stem Cell Marker Gene Expression Profiling

Signosis’s Human stem cell marker cDNA plate array was used to examine stem cell related gene expression on spheroid cells and parent cells according to the manufacturer’s protocol. Briefly, total RNA isolated from cells by Tri Reagent (Molecular Research Center, Inc.) was reverse transcribed into cDNA, which was hybridized with gene-specific oligonucleotide pre-coated in individual wells. The expression level of genes was detected by chemiluminescent signals by a plate reader.

### Quantitative PCR

Total RNA was exacted from spheroid or non-spheroid cells using Qiagen’s RNeasy Mini Kit per manufacture’s procedure and reverse transcribed into cDNA by iScript cDNA Synthesis Kit from Bio-Rad. Quantitative real-time PCR was performed using Bio-Rad’s iQ SYBR Green Supermix per company’s protocols in a iCycler iQ system also from Bio-Rad. Real-time PCR primer sequences are shown in Table 1. Thermal cycling was performed by an initial denaturing step at 95°C for 3 min followed by 40 cycles of denaturation at 95°C for 10 sec, then 60°C for 1 min for annealing and data collection. Melt-curve analysis was performed immediately after the amplification protocol under the following conditions: 80 cycles of 0.5°C increments (10 sec each) beginning at 57.5°C (data collection step). Each experiment was performed in triplicate, with normalization to the ribosomal protein L4 (RPL4) gene as an internal control and target gene expression level was calculated by ΔΔC_t_ method using Bio-Rad’s iQ5 quantitative PCR analysis software.

**Table 1:** Quantitate real time PCR primers used in this study.

	Type	Sequence
Notch1		
ACC# NM_017617.3	Forward	GGGACCAACTGTGACATCAA
	Reverse	GTAGCCACTGGTCATGTCTTT
CD34		
ACC# NM_001773.2	Forward	TAGCCTGTCACCTGGAAATG
	Reverse	TGCCTTGATGTCACTTAGGATAG
Cdcp1		
ACC# AF468010.1	Forward	AAGGACACAGACATTCCCTTAC
	Reverse	GTCTCAGTGCCCTGCTTTAT
Nanog		
ACC# NM_024865.2	Forward	TCCTGAACCTCAGCTACAAAC
	Reverse	GCGTCACACCATTGCTATTC
Myc		
ACC# NM_002467.4	Forward	CTTCTCTGAAAGGCTCTCCTTG
	Reverse	GTCGAGGTCATAGTTCCTGTTG
RPL4		
ACC# AK294113.1	Forward	GGCTACAAGAAGACCAAGGAA
	Reverse	CTCATTCGCTGAGAGGCATAG

### ALDH Activity Analysis

The ALDEFLUOR kit (StemCell Technologies, Vacouver, Canada) uses a fluorescent substrate which accumulates within cells after oxidized by ALDH enzyme. Cells in a concentration of 2×10^5^/ml were stained by 5 µl ALDEFLUOR reagent at 37°C for 45 min. In each experiment, a sample of cells was stained under identical conditions with specific ALDH inhibitor diethylaminobenzaldehyde (DEAB) as negative control for setting up flow cytometry gate. The BioVision ALDH Activity Assay kit (BioVision) quantifies ALDH enzymatic activity by absorbance reading at 450 nm. Acetaldehyde is oxidized by ALDH generating NADH which then reduces a colorless probe to a colored product. Spheroid cells or parental cells (1×10^6^) were lysed by 200 µl ALDH Assay Buffer and 10 µl of the cell lysate were used for the assay. OD450s were read at 10 min and 1 hr intervals and ALDH activities were calculated according to manufacturer’s protocol. All tests were done in duplicate.

### Proliferation Assay

Parental and spheroid EOC cells were cultured in complete medium (CM, 10%FBS+RPMI) or unsupplemented RPMI for 24 h. Then the same amounts of cells were seeded in tissue culture plate with CM, half of the medium was changed every 3 days and cell growth was detected by CellTiter-Glo Luminescent Cell Viability Assay as described [18] (Promega Corp). The proliferation assays were done in triplicate.

### Migration Assay

Parental and spheroid EOC cells were treated with unsupplemented RPMI for 24 h. In a transwell plate, 2×10^5^/well cells were put into insert with 0.2%BSA-RPMI medium, the chamber has no cells except RPMI medium with 2.5% FBS. After culture for 1, 2, and 3 days, the numbers of cells that passed through membrane as well as cells fall into chamber were calculated under microscope after 10% formaldehyde fixing for 20 min followed by 0.1% crystal blue staining for another 20 min both at room temperature (RT). All tests were done in duplicate.

### Wound Healing (Scratch) Assay

Parental and spheroid EOC cells were cultured in CM for 24 h. Then cells were seeded into 6-well plates to 80–90% confluence and the cell monolayer was scratched in a straight line with a 200 µl pipette tip to create a “scratch”. Debris was removed with PBS and then the culture was re-fed with fresh medium. Images were taken at 0 and 24 hr after the scratch to calculate the cell migration rate.

### Invasion Assay

Parental and spheroid EOC cells were treated with RPMI only medium for 24 h. In a transwell plate, 40000/well cells were put into insert which is coated with matrigel in 0.2%BSA-RPMI medium, the chamber has no cells except RPMI medium with 5% FBS. After culture for 1, 2, and 3 days, inserts were collected and the surface of membranes were cleaned, cells in matrigel were fixed by 10% formaldehyde for 20 min RT and the numbers of cells that invaded into matrigel were determined after staining with 0.1% crystal blue 20 min RT. All experiments were done in duplicate.

### Anchorage-independent Growth Assay

Five hundred cells were seeded in triplicate in 6-well plates containing a top layer of 0.3% soft agar and a 0.5% agar base in DMEM, 10% FBS. Twenty-four hours later, the average number of cells seeded per field was determined by counting cells in 5 different fields under the light microscope. Colonies formed (>0.1 mm in diameter) after 3 weeks of growth in soft agar were counted; 10 different fields were quantified per well and the average number of colonies per field was calculated. The AIG (anchorage-independent growth) index was expressed relative to the number of cells seeded.

### Chemotherapy Resistance Assay

Spheres were dissociated by trypsinization and pipetting and cells were seeded at 5,000 cells per well (96-well plates; Corning) in 200 µL CM medium. All cells were treated for 24 h with 0 to 6 µg/mL Cisplatin (BD Biosciences) or 0 to 18 µg/mL Paclitaxel (Sigma; n = 5 per drug dose). Relative cell numbers were determined by CellTiter-Glo Luminescent Cell Viability Assay [18]. Dose-response experiments were performed in duplicate. Percentage cell survival is expressed relative to untreated control. Spheroid cell drug cytotoxicities were compared after 48-h treatment with Cisplatin (4 µg/mL). After drug treatments, cells were incubated for 8 days in drug-free CM medium and cell colonies were examined under microscopy.

### Clonogenic Survival Assay

Cells were treated with various amounts of Cisplatin for 24 h. After removing the drug, cells were washed with PBS, harvested and 300 of surviving cells/well were re-fed with CM medium in 6-well plate. 10–14 days later, colonies were stained with 0.1% crystal violet and counted.

### Immunohistochemistry

Tumor specimens were fixed with buffered formalin and embedded in paraffin. Sections (5 µm) were placed on glass slides, heated at 60°C for 20 min, and then deparaffinized with xylene and ethanol. For antigen retrieval, tumor specimens mounted on glass slides were immersed in preheated antigen retrieval solution (DAKO high pH solution; DAKO, Carpinteria, CA) for 20 min and allowed to cool for 20 min at room temperature. After the inactivation of endogenous peroxidase, purified specific monoclonal antibody CA125 (Ov 185∶1, Novocastra) (1∶200 dilution) and CK7 (Dako) (1∶20 dilution or 75 µg/ml) were then added, and incubated overnight at 4°C. The primary antibody was detected with a biotinylated anti-mouse IgG (DAKO). Diaminobenzidine tetrahydrochloride was then added for development for 10 min, followed by counterstaining with hematoxylin solution.

### Biochemical Analyses

The use of [U-^13^C_6_] glucose tracer in combination with mass spectrometry allows the evaluation of metabolic flux through the main pathways facilitating energy production and biosynthetic metabolism of the cell. Normal ovarian cells (RPNLOv78) were kindly provided by Dr. T Pejovic [19]. The cells were cultured in 1∶1 mixture of RPMI and DMEM (glucose-free) +10% FBS +10 ug/ml Insulin +10 ng/ml EGF+0.1% Gentamicin+ [U-^13^C_6_]glucose (180 mg/ml). Parent cells (RP-OV17534) were cultured in RPMI (glucose-free) +10%FBS+[U-^13^C_6_]glucose (180 mg/ml). RP-OV17534 spheroid cells were cultured in DMEM/F12 (glucose-free) +N2 Supplement-A +EGF 10 ng/ml +bFGF 10 ng/ml+[U-^13^C_6_]glucose (180 mg/ml). After 24 hrs of the incubation, cells were centrifuged (1,500 rpm for 5 minutes) and incubation medium and cell pellets were obtained. Glucose and lactate incubation medium concentrations were determined as previously described [20]. Lactate from the cell culture media was extracted by ethyl acetate after acidification with HCl. Lactate was derivatized to its propylamide-heptafluorobutyric form and the *m/z* 330 and 331 (carbons 2–3 of lactate, chemical ionization) was monitored for the detection of *m2* (^13^C double-labeled lactate) and *m3* (triple-labeled lactate) for the estimation of pentose cycle activity versus anaerobic glycolysis [21]. Glutamate was separated from the medium using ion-exchange chromatography [22]. Glutamate was converted to its *n*-trifluoroacetyl-*n*-butyl derivative and the ion clusters *m/z* 198 (carbons 2–5 of glutamate, electron impact ionization) were monitored. ^13^CO_2_ release was measured by a Finnegan Delta-S ion ratio mass spectroscope and was used to estimate glucose carbon utilization through oxidation by the cell lines [23]. Mass spectral analyses were carried out by three independent automatic injections by the sampler and accepted only if the standard sample deviation was <1% of the normalized peak intensity.

### Data Analysis and Statistical Methods

Statistical analyses were done using the parametric unpaired, two-tailed independent sample *t* test with 99% confidence intervals.

## Results

### Generation of Spheroid Cells from Primary Ovarian Cancer Specimens and Established EOC Cell Lines

**Figure 1:**
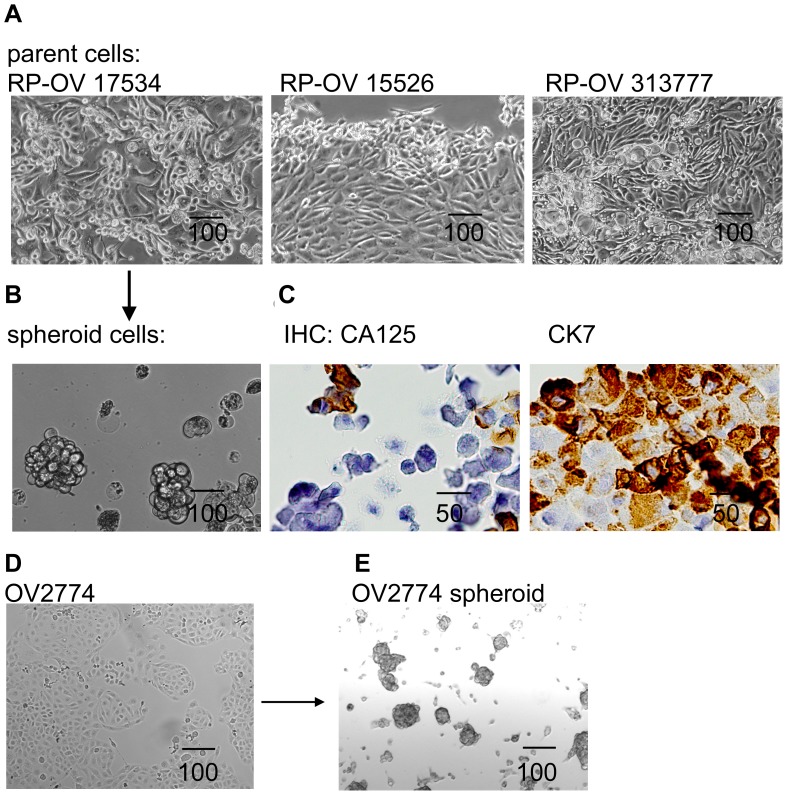
Spheroid cells were generated from primary and established EOC cells. (A) Three primary cell lines generated from EOC patients show epithelial cells morphology. (B) One of primary EOC cell line generated 3-D independent, self-renewing spheroid cells under stem-cell selection media. (C) Primary cell lines express ovarian carcinoma marker CA-125 and epithelial marker CK-7, as shown by IHC for RP-OV17534. (D) EOC cell line OV2774 is able to generate OV2774 spheroid cells (E). All scale bar unit in this figure and following figures are in µm.

Stable cell lines were established successfully from 3 of 30 (10%) cultures initiated from primary EOC specimens over a period of 2 years. RP-OV15526 was derived from solid tumor while RP-OV17534 and RP-OV313777 were derived from EOC ascites. These cells have been cultured *in vitro* for 465, 312, and 125 days with 68, 65, and 17 passages, respectively. All lines were from late-stage (IIIC or IV) ovarian serous adenocarcinoma cancer patients. Confluent monolayer of primary human EOC cells depicted typical epithelial cobblestone morphology with 3-dimensional growing upwards in cell-condensed areas (**Fig. 1A**). Examination of epithelial markers CK-7 and CA-125 further confirmed the epithelial nature of these primary cell lines (**Fig. 1C**). In order to generate spheroid cells, ovarian cancer cell line OV2774 or primary EOC cells were enzymatically dissociated and inoculated on ultra-low attachment culture plates in serum-free medium with EGF, bFGF, and N2 supplement-A. In this culture condition some cells died from serum starvation, while others were forced into suspension and formed aggregates. Three weeks after plating, some aggregates compacted into spheres which could not be dissociated by pipetting. Some of the spheres also aggregated to form sphere clusters. Floating spheres and clusters were dissociated by pipetting, and replated twice a week, with the resulting cells generating secondary spheres, appearing as distinct prototypical spheroids (**Figs. 1B, 1E**), similar to those found in patient ascites [5], [6]. Using this approach, we obtained sustainable spheroids from established cell line OV2774 (**Figs. 1D and 1E**) and primary EOC cells RP-OV17534 (**Figs. 1A and 1B**) under stem-selective conditions. We have cultured the OV2774 and RP-OV17534 cells as spheroids for 6 months, demonstrating the self-renewing ability of the spheroid cells.

### Spheroid Cells Tumorigenicity and Metastasis in Immune-deficient Mice

**Figure 2:**
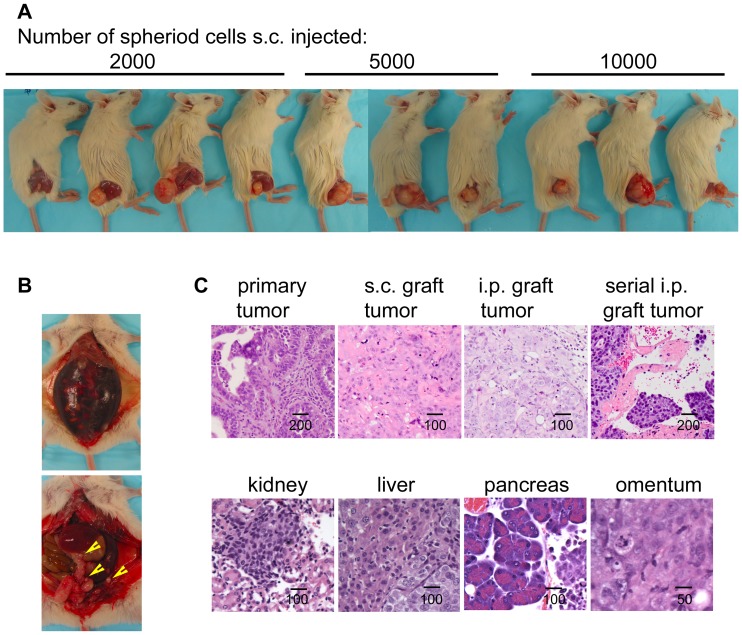
RP-OV17534 Spheroid cells generated tumors in immune-deficient mice. (A) Different amount of spheroid cells s.c. injected into SCID mice formed tumors. (B) Spheroid cells i.p. injected into SCID mice formed bloody ascites and tumors in different organs as indicated by arrows. (C) Representative H&E staining sections (upper panel) shows RP-OV17534 primary tumor, subcutaneous graft tumor from spheroids, intraperitoneal graft tumor from spheroids, and serial intraperitoneal graft tumor from SCID mouse ascites. Histological analysis (lower panel) shows frequent metastasis of tumor cells into various organs of spheroid cells recipients.

Next, we investigated the tumorigenicity of sphere-forming cells. We examined whether exponentially smaller numbers (compared with parental cancer cells) were capable of tumorigenesis, as previously shown for other epithelial cancer initiating cells (CICs) [24]–[28]. Spheroid cells or corresponding parental bulk tumor cells were injected s.c. into right legs of SCID mice. With injections of only 2,000 cells per mouse, spheroid cells were tumorigenic in 4 of 4 SCID mice for RP-OV17534 spheroid cells and 2 of 2 for OV2774 spheroid cells, as evidenced by palpable tumors at the injection site (**Fig. 2A** **;** **Table 2**). The median tumor latency time in this cohort was 31 to 90 days for RP-OV17534 spheroid and 50 to 57 for OV2774 spheroid, similar to or less than CICs of other malignancies [24]–[27]. Correspondingly, injections of 5,000 and 10,000 RP-OV17534 spheroid cells were also tumorigenic in three of three mice with shorter tumor latencies (**Fig. 2A** **;** **Table 2**). Without non-adherent spheroid selection, bulk tumor cells failed to form tumors even at 40,000 cells for RP-OV17534 engraftment (**Table 2**). All subcutaneous xenograft tumors derived from spheroid cells were categorized as serous adenocarcinomas of moderate/poor differentiation (grade 2/grade 3), similar to the parental primary patient tumors (H&E stained sections; **Fig. 2C**). No architectural/cytologic differences were observed between primary and graft tumors.

**Table 2:** In vivo tumorigenicity of ovarian cancer spheroid cells.

*Cell type*	*Injection cell type*	*Injection site*	*Cell dose* a	*Tumor formation* b	*Latency* c *days*
Primary	Spheroid	S.c.	2000	4/4	31/38/42/90
EOC		S.c.	5000	3/3	40/52/54
RP-OV		S.c.	10000	3/3	31/37/38
17534	Parental	S.c.	5000	0/2	–
		S.c.	10000	0/2	–
		S.c.	40000	0/2	–
	Spheroid	I.p.	10000	4/4	75/80/80/84
		I.p.	20000	3/3	59/63/63
		I.p.	40000	3/3	54/54/63
	Parental	I.p.	20000	0/2	–
		I.p.	40000	0/2	–
		I.p.	60000	0/2	–
		I.p.	80000	0/3	–
		I.p.	1.7×10^7^	1/1	92
		I.p.	2×10^7^	3/3	105
EOC cell	Spheroid	S.c.	2000	2/2	50/57
line	Parental	S.c.	2000	1/2	57
OV2774	Spheroid	I.p.	5000	1/1	126
		I.p.	15000	1/1	85
		I.p.	20000	1/1	126
		I.p.	300000	1/1	79
	Parental	I.p.	20000	0/1	–
		I.p.	40000	0/1	–
			1.2×10^7^	1/1	50
			2×10^7^	1/1	38

^a^ Number of cells per injection.

^b^ Number of tumors formed per number of injection.

^c^ The time from injection to the first appearance of a palpable tumor or ascites.

S.c.: subcutaneous; I.p.: intraperitoneal.

A major limitation of studies of CICs is engraftment into non-native microenvironments [29], [30]. To establish that CICs faithfully recapitulate the well-established progression of ovarian cancer in its native setting, the experiments were conducted with intra-peritoneal (i.p.) injections. The i.p. injection of only 10,000 cells per mouse of RP-OV17534 spheroid cells resulted in development of bloody ascites in 4 of 4 SCID mice (**Fig. 2B** **;** **Table 2**), with tumor latencies of 75 to 84 days, similar to or less than CICs of other malignancies [17]. Correspondingly, i.p. injections of 20,000 and 40,000 spheroid cells were also tumorigenic in three of three mice with shorter tumor latencies (**Table 2**). Without non-adherent spheroid selection, bulk tumor cells failed to form bloody ascites and tumors even at 80,000 cells per injection, whereas one of one and 3 of 3 mice i.p. injected respectively with 1.7×10^7^ and 2×10^7^ parental primary EOC cells were tumorigenic, albeit with extended latency (92–105 days; **Table 2**). I.p. injection of sphere-forming cells resulted in development of bloody ascites and peritoneal metastasis to the omentum, liver, colon, stomach, and kidney (**Fig. 2B**), and intraperitoneal tumor histology similar to both subcutaneous xenograft and primary patient tumors (**Fig. 2C**). Consistent with the results for RP-OV17534, we also observed higher tumorigenicity of OV2774 spheroid cells compared to their parent cells, in similar i.p. engraftment animal experiments using OV2774 derived cells (**Table 2**).

Another essential criterion for CICs is their ability to serially propagate tumors in consecutively engrafted animals [31]. To examine this definitive stemness characteristic, serial engraftments of xenografts were performed. 2×10^4^ of spheroid cells were i.p. injected into SCID mice and ascites developed as expected in 3 out of 3 mice in approximately 60 days. Transplantation of 8×10^4^, 1×10^5^, and 5×10^5^ such ascites cells into SCID mice resulted in ascites and tumors, with a latency significantly shorter than the parental patient tumor cells (66/63/49 days respectively for passage 2 xenografts versus no tumor development for parent tumor cells of 8×10^4^ transplantation in 0 out of 3 SCID mice). The pathology of the omental mass was similar to that of s.c. tumors (**Fig. 2C**). These results indicate that sphere-forming cells are more tumorigenic than their parental tumor cells, demonstrating that a highly tumorigenic subpopulation of cells are present within the sphere-forming cells, and may reside within ovarian neoplasms. Since RP-OV17534 spheroid cells and OV2774 spheroid cells demonstrated similar characteristics, the following results are shown for RP-OV17534 derived cells with similar observations for OV2774 cells.

### Ovarian Tumor Sphere-forming Cells Express Stem Cell Genes

**Figure 3:**
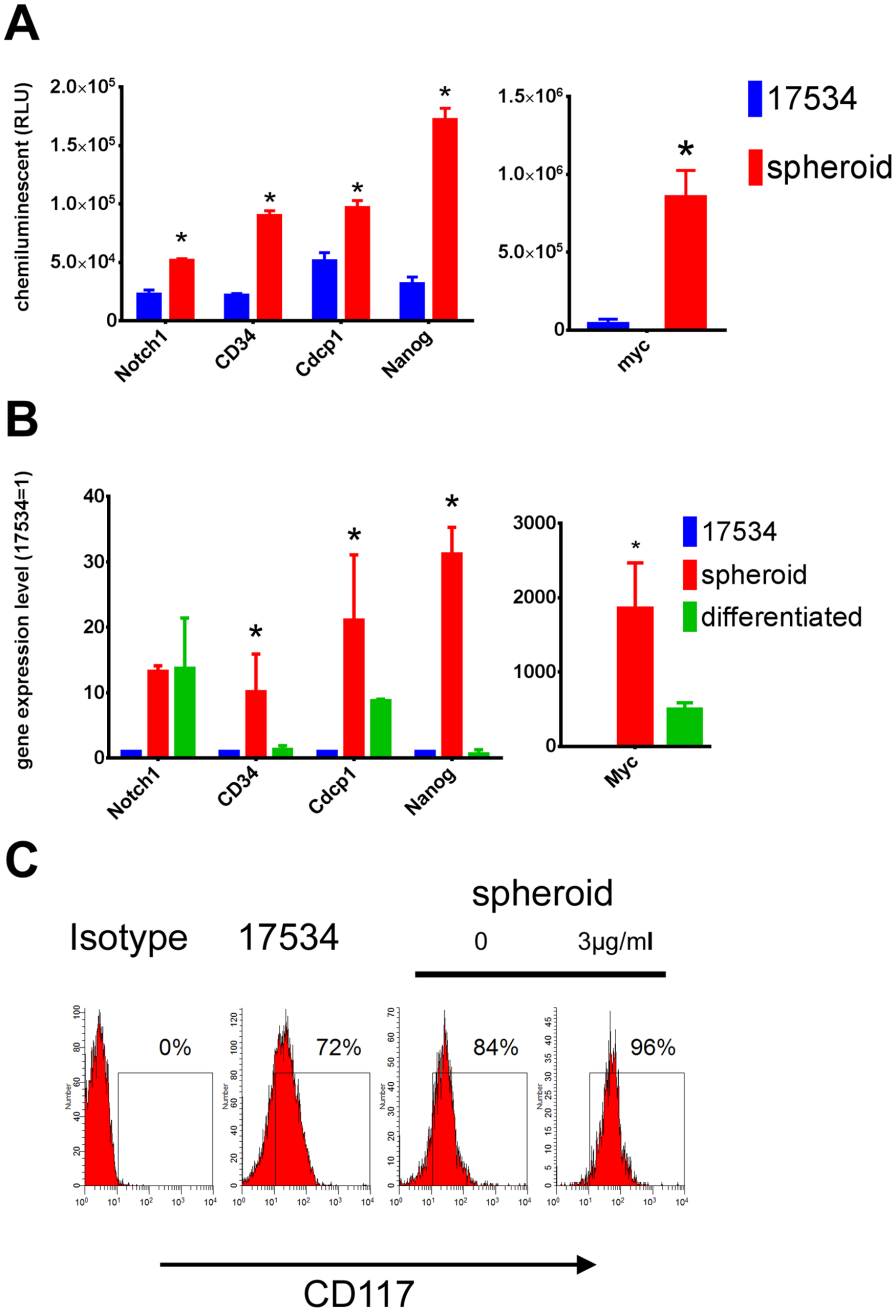
Spheroid cells overexpressed stem cell genes. (A) Total RNA isolated from spheroid cells and parent cells were examined for their gene expression by a human stem cell markers cDNA array and stem cell-genes showed higher expression levels in spheroid cells to non-spheroid cells. (B) The overexpression levels of Notch1, Nanog, Cdcp1, CD34, and Myc in spheroid cells compared to non-spheroid cells were confirmed by quantitative real-time PCR. These stem cell-genes’ expression were lost or down-regulated when spheroid cells were differentiated by culturing in CM without growth factors for 14 days. Expression levels are represented as fold changes compared to these of non-spheroid cells. (C) FACS examination of CD117 expression on spheroid cells and parent cells showing high CD117 expression in spheroid cells. Some spheroid cells were treated with Cisplatin for 24 hrs before surface staining by CD117 Abs. Error bars: SD, N = 3. *: p<0.05.

To examine the expression of gene specific to embryonic stem cells, total RNA from spheroid cells and parent cells were analyzed by a Human Stem Cell Marker cDNA Plate Array. Among 32 genes tested, spheroid cells upregulated Notch1, Nanog, Cdcp1, CD34, and Myc expression (**Fig. 3A**). Each of these genes is essential for developmental processes (embryogenesis, neurogenesis, stem cell expansion, and hematopoiesis [32]–[34]). These results were confirmed by quantitative real-time PCR data. The expression levels of Notch1, Nanog, Cdcp1, CD34, and Myc in spheroid cells were approximately 10 to 2000 fold higher than that of non-spheroid cells detected by real-time PCR (**Fig. 3B**, red bar). When spheroid cells were cultured in CM without growth factors, a differentiating condition, floating cells adhered and grew into epithelial cells. Next we compared the same sets of gene expression level by real-time PCR in spheroid cells, culturing in either stem cell-selective or differentiating conditions for 14 days. Expression of CD34 and Nanog were approximately 1 fold of that of non-spheroid cells, Cdcp1 and Myc decreased to 9 and 500 fold, respectively. Notch1 expression level in spheroid cells stayed at 12 fold of that of non-spheroid cells after culturing in CM for 14 days (**Fig. 3B**, green bar). Stem cell factor receptor CD117 (c-kit) has been reported in previous studies on ovarian cancer tumor progenitors [17], [35]. Therefore, we examined CD117 expression by FACS in these spheroid cells. Consistent with previous reports, we also detected CD117 up-regulation in spheroid cells compared with parent cells. Since a large number of spheroid cells killed by chemotherapy drugs were differentiated non-stem cells, whereas stem-like cells survived (see below), we found that CD117 expression further increased in spheroid cells after cisplatin treatment for 24 hrs (**Fig. 3C** **)**. These data indicate that spheroid cells from ovarian cancer overexpress stem cell genes under stem cell-selective conditions and lose or decrease these gene expressions under differentiation conditions.

### Increased ALDH Activity in Spheroid Cells

**Figure 4:**
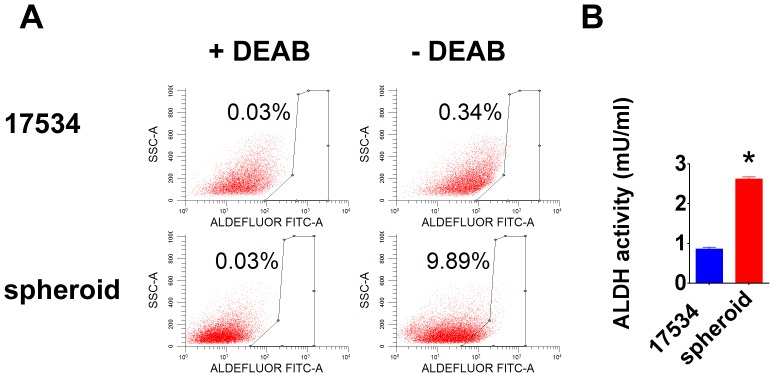
Increased ALDH activity in spheroid cells. (A) The ALDEFLOUR kit labels the population with a high ALDH enzymatic activity in spheroid cells. An aliquot of each sample of cells was treated with ALDH inhibitor DEAB as negative control for setting FACS gate. (B) The BioVision ALDH colorimetric assay detected enhanced ALDH activity in spheroid cell lysate. Error bars: SD, N = 2. *: p<0.05.

ALDH plays a vital role in cellular detoxification. Recent studies show that increased ALDH activity leads to several types of malignancies, serves as a cancer stem cell marker and correlated with poor prognosis [36]–[39]. Our ALDEFLUOR assay showed more ALDH^+^ cells in spheroid cells in the absence of ALDH inhibitor DEAB (**Fig. 4A**). The increased ALDH function in spheroid cells was confirmed by an ALDH activity colorimetric assay, showing significant enhanced activity in spheroid cells (**Fig. 4B**).

### Spheroid Cells have High Proliferation and Migration Potential than their Parental Cells

**Figure 5:**
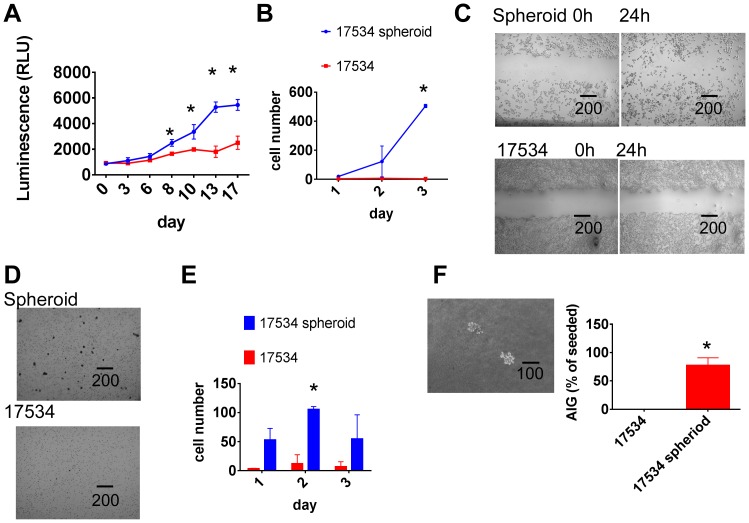
Spheroid cells show a more aggressive growth pattern. (A) Spheroid cells had higher proliferation potential than their parent cells. Both spheroid cells and parental cells were cultured in CM for 24 h, then 5000/well cells were cultured in CM in 96 well plates. Half of media was changed every 3 days. Cell proliferations were detected from day 0 to day 17 by CellTiter-Glo Luminescent Cell Viability Assay. (B) Spheroid cells had higher migration ability than parent cells. Parental and spheroid EOC cells were treated with RPMI only medium for 24 hrs. In a transwell plate, 2×10^5^/well cells were put into insert with 0.2%BSA-RPMI medium, the lower chamber contains no cells except RPMI medium with 2.5% FBS. After culture for 1, 2, and 3 days, the numbers of cells that passed through membrane as well as cells fall into chamber were calculated under microscope. (C) Spheroid cells recovered ‘scratch’ made by 200-µL pipette tip after 24 hours more efficient than their parent cells. Experiments were repeated three times with similar results. (D) More spheroid cells invaded into matrigel than parent cells as shown by microscopy. Spheroid cells and parental cells were treated with serum-free medium RPMI for 24 h. In a transwell plate, 40000/well of cells were put into insert coated with matrigel with 0.2% BSA, while the chamber had only media with 5% FBS, after culture for 1, 2, and 3 days, The number of cells invading into matrigel were counted after 0.1% crystal blue staining. (E) Invasion tests were done in duplicate and average and SD were shown. *,p<0.01. (F) Spheroid cells formed colonies in top agar detected in anchorage-independent growth assay (AIG) as shown under microscopy. 500 cells were seeded in 6 well plates containing a top layer of 0.3% soft agar and 0.5% agar base in DMEM, 10% FBS. 24 hrs later, the number of cells seeded was determined by counting cells under the light microscope. After 3 weeks of growth in soft agar, the number of colonies (Colonies >5 cells) were counted; 10 different fields were quantified per well and the average number of colonies per field was calculated. The AIG index was expressed relative to the number of cells seeded. More than 70% of seeded spheroid cells formed colonies while none of seeded parent cells formed colonies. Experiments were repeated twice with similar results. *,p<0.01. Error bars: SD.

The relationship of spheroid formation and cell growth potential was examined in a kinetic cell proliferation assay. Increased cell proliferation was demonstrated in spheroid cells compared with parent cells. More cells appeared at day 6 and continued increasing to day 17 (**Fig. 5A**). Since all cell cultures were maintained at identical growth conditions in the same culture milieu, their varying growth kinetics could be attributed to cell intrinsic mechanisms.

Cell motility is one of the factors that contribute to tumor cell invasion [40]. To detect the migration kinetics of spheroids versus non-spheroid cells, parental and spheroid EOC cells were studied in a migration assay. Very few parental cells migrated while significant amount of spheroid cells passed through transwell membrane. The difference started to show on day 2 and continued on day 3 (**Fig. 5B**). The observation that spheroid cells migrated quicker than their parental cells was further confirmed by a wound healing assay. As shown in **Figure 5C**, more spheroid cells migrated into the scratch than parental cells. The data also suggest spheroid cells may utilize different wound healing pathways.

### Ovarian Tumor Spheroid Cells Showed Invasion Abilities and Anchorage-independent Growth

To study the relationship between spheroid formation and cell invasion, spheroid cells and parental cells were tested in an invasion assay. In this assay, cells need to break down matrigel while in migration assay cells just moved through a membrane. The invasion assay mimics the *in vivo* situation more closely. As shown in **Figure 5D**, very few parent cells invaded into matrigel while significantly more spheroid cells invaded into matrigel after 3 days of culture. Spheroid cells had higher invasion ability than their parental cells detected on day 1, 2, and 3 (**Fig. 5E**). Since spheroid cells show increased proliferation ability after 6 days of culture in proliferation assay, it is unlikely that the superior invasion ability of spheroid cells is merely due to their higher proliferation property. This *in vitro* invasion potential is consistent with the observed *in vivo* spheroid cells tumorigenicity as shown in **Fig. 2**. To further confirm the aggressive properties of spheroid cells, we next investigated their tumorigenic potential using the anchorage-independent growth (AIG) assay. **Fig. 5F** shows that parent cells could not form colony in soft agar while spheroid cells produced significant numbers of colonies, providing additional evidence for the increased tumorigenic attributes of spheroid cells.

### Spheroid Cells are Resistant to Conventional Chemotherapies

**Figure 6:**
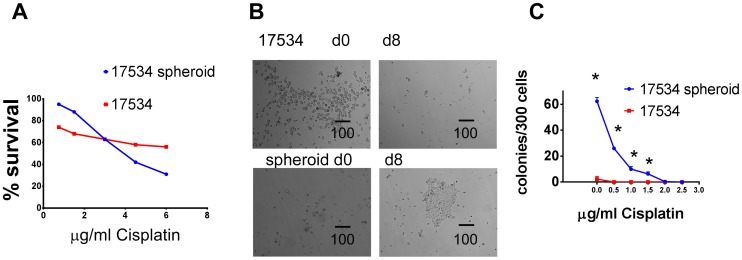
Spheroid cells are resistant to chemotherapy *in vitro*. (A) More spheroid cells were killed by Cisplatin than parent cells. The same numbers of spheroid and parent cells were cultured in CM with indicated amount of Cisplatin for 2 days and numbers of cells from different time points were determined. (B) More parent cells survived after Cisplatin treatment for 48 h (Left). However, 8 days later, only spheroid cells were growing while parent cells disappeared (Right). (C) Spheroid and parent cells were treated with indicated amount of Cisplatin for 24 h, washed, and 300 of survived cells/well were cultured in CM for another 10 days, cell colonies were shown after crystal violet staining. The same clonogenic survival assays were repeated twice with similar results. Colonies formed (>5 cells) after 10 days of growth were counted and average and SD were shown. *,p<0.05.

To determine whether spheroid cells possess a cancer stem cell chemoresistant phenotype, spheroid cells and parent cells were treated in the same serum-free culture medium for 24 h. After which, 5000/well of cells was treated by various amounts of Cisplatin in CM. Numbers of cells were determined by CellTiter-Glo Luminescent Cell Viability Assay at different time points. More spheroid cells were killed by Cisplatin at concentrations above 4 µg/mL on day2 while only few non-spheroid cells were dead at the same concentrations, suggesting that spheroid cells are initially more sensitive to Cisplatin than non-spheroid cells due to their proliferation potential (**Fig. 6A**). However, when cells treated with Cisplatin for 48 h were washed and cultured in CM for another 8 days, spheroid cells formed more colonies than non-spheroid cells (**Fig. 6B**). These results suggested that a large number of spheroid cells killed by Cisplatin are differentiated non-stem cells while the small number of cells that survived the chemotherapy treatment are resistant stem-like cells with more proliferative potential compared to non-spheroid cells once chemotherapy treatment was removed. To confirm this hypothesis, we investigated clonogenecity of residual cells after Cisplatin treatment. Spheroid cells and parent cells were treated with increasing concentration of Cisplatin for 24 hr, washed and 300/well of treated cells were cultured in 6-well-plate for another 10 days, the numbers of colonies (>5 cells) were counted under microscopy. Untreated spheroid cells generated many colonies while parent cells formed significantly less colonies, consistent with our chemotherapy survival assays. As the Cisplatin concentration increased, the number of colonies decreased. However, spheroid cells still formed significantly more colonies than parent cells after Cisplatin treatment (**Fig. 6C**).

### Metabolism Studies Reveal Spheroid Cells’ Resistance to Oxygen Deprivation

**Figure 7:**
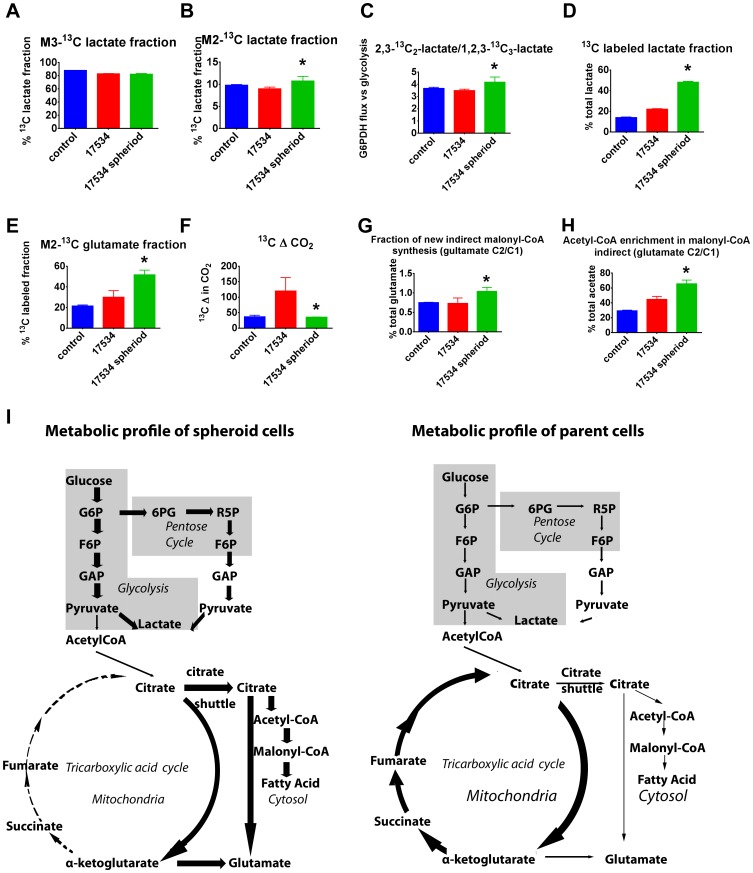
Spheroid cells show a hypoxia-resistant metabolism profile. Metabolic profiles of a normal ovarian cell line RPNLOv78 as control, EOC cells RP-OV17534, and spheroid RP-OV17534 cells cultures in the presence of [U-^13^C_6_]glucose for 24 hrs (n = 3) showing (A) percentages of m3-^13^C lactate derived directed from glycolysis; (B) percentages of m2-^13^C lactate derived from pentose cycle; (C) ratio of m2 to m3 lactate; (D) percentage of ^13^C-labeled lactate of total lactate; (E) percentages of ^13^C-double-labeled glutamate in total ^13^C-labeled glutamate; (F) ^13^CO_2_ release from [U-^13^C_6_]glucose; (G) The fraction of newly synthesized palmitate and the ^13^C enrichment of acetyl units (H). Error bars: SD; *,p<0.01, compared with parental EOC cells. (I) EOC spheroid cells show increased anaerobic glycolysis, increased direct glucose oxidation in the pentose cycle, but low TCA cycle carbon flux, low anaplerotic flux, and *de novo* fatty acid synthesis. In contrast, parent EOC cells show increased TCA cycle and high anaplerotic flux. G6P, glucose-6-P; F6P, fructose-6-P; GAP, glyceraldehyde-3-P; 6PG, 6-P-gluconate; R5P, ribulose-5-P. Various thickness of lines represent different strengths of metabolic flux.

Cancer cells adopt an alternative metabolic pathway and increase their glycolytic activity through a process called the Warburg effect [41]. The Warburg effect is necessary for cancer cells to resist oxidative stress and to adapt to hypoxic conditions [42]. Since glycolysis could play an essential role in tumorigenesis during both the immortalization and transformation steps [43], we investigated the dynamic metabolic flux of spheroid cells. Lactate secreted into cell culture medium can be used to measure label incorporation into the three-carbon metabolite pool. Direct [U-^13^C_6_] glucose catabolism through glycolysis results in m3 lactate, whereas passage through the oxidative and nonoxidative branches of the pentose phosphate pathway of glucose results in m2 lactate. As expected, the majority of lactate is produced via glycolysis in all tumor cell cultures (**Fig. 7A**). Spheroid cells showed significant increase in oxidizing glucose directly in the pentose cycle (**Fig. 7B**). Metabolism flux comparison between Glucose-6-P Dehydrogenase (G6PDH) and glycolysis revealed increased pentose cycle flux and NADPH production in spheroid cells, indicating increased acetate use for *de novo* fatty acid synthesis instead of complete oxidation in spheroid cell cultures (**Fig. 7C**). The ^13^C label enrichment of lactate from glucose in spheroid cells showed a more than two fold increase compared with their parent cells (**Fig. 7D**). The ^13^C isotopomer distribution showed a significant increase in anaerobic glycolysis and defective aerobic glucose metabolism in spheroid cells. This is indicative and important marker of decreased tricarboxylic acid (TCA) cycle flux and energy production via complete glucose/substrate oxidation. TCA cycle anaplerotic flux was measured using the equilibrium between glutamate and α-ketoglutarate. The large increase in glutamate labeling in two carbon positions with ^13^C indicated a defective TCA cycle and limited turnover of ketoglutarate into succinate and fumarate (**Fig. 7E**). This indicates a complex-II defect in spheroid cell cultures. Consistently, ^13^C-labeled carbon dioxide release from glucose was significantly decreased in spheroid cells (**Fig. 7F**), which also indicated that spheroid cells have defects in complete glucose oxidation in the TCA cycle. ^13^C-gultamate and acetyl-CoA are the transported products of the citrate shuttle from mitochondria to the cytosol for *de novo* fatty acid synthesis, fatty acid chain elongation and glutamate recycling. Glutamate labeling in the cytosol is similar to that of malonyl-CoA and fatty acids up to palmitate (C:16) via substrate sharing through the citrate shuttle. ^13^C-acetate incorporation from substrate into fatty acids and rate of new fatty acid synthesis, which corresponds with the fraction of newly synthesized glutamate, was measured by glutamate labeling in the cytosol. The fraction of newly synthesized fatty acid increased significantly in spheroid cells (**Fig. 7G**), and ^13^C enrichment of acetate for fatty acid synthesis also showed significant increase (**Fig. 7H**). Spheroid cells had significant increase in malonyl-CoA synthesis and fatty acid *de novo* synthesis and breakdown via peroxisomal beta oxidation. The large increase of glucose derived acetate used for malonyl-CoA synthesis also indicated severe defect in TCA cycle energy production and turnover. The main energy production and biosynthetic metabolism pathways for both spheroid cells and parent cells are summarized in Figure **7I**.

## Discussion

There are both single cells and spheroid cells in EOC tumors and ascites [44], [45]. Previous research in ovarian cancer has focused mainly on the metastatic behavior of single cells. Currently, there is data to demonstrate that spheroids are capable of tumorigenesis *in vivo*, and have a reduced response to chemotherapeutic drugs *in vitro* [46]–[48]. In order to study EOC pathogenesis, we cultured EOC primary cells under stem cell-selective conditions and generated anchorage-independent, self-renewing spheroids morphologically similar to spheroids isolated from patient ascites [5], [6]. These spheroid cells expressed numerous stem cell markers and were sustainable indefinitely under stem cell-selecting conditions. The spheroid cells had shorter doubling time than their parental cells and showed additional attributes that are distinct from their parent cells. Strikingly, as few as 2000 spheroid cells from primary tumor cells engrafted into immune-deficient mice and allowed full recapitulation of the original tumor, whereas >10^5^ parent tumor cells remained non-tumorigenic. These spheroid cells also could serially propagate tumors of identical phenotypes *in vivo*. In order to address concerns regarding engraftment into non-native microenvironment [29], [30], we i.p. injected EOC spheroid cells into SCID mice which resulted in a pathology essentially identical to the human malignancy, with formation of bloody ascites and extensive peritoneal dissemination. Although there is a long history of transplanting human tumor cells into immune-deficient mice, the strong host resistance in these mice allowed only the most aggressive tumors and cell lines to be engrafted. Our *in vivo* engraftment experiments not only indicated that spheroid cells are highly tumorigenic but also that EOC is hierarchically organized so that only a subset of cells-cancer stem cells- possess the potential to initiate and sustain tumor growth.

Cell motility is one of the factors that contribute to tumor cell invasion. Indeed, spheroid cells showed higher migration ability than their parent cells both in migration and wound healing assays. Importantly, spheroid cells had higher invasion ability than parent cells in invasion assays. They also showed 3-D growth in anchorage-independent growth assays. These results are consistent with previous reports [5], [7], [49], [50] indicating that spheroids could adhere to, migrate on and infiltrate into other monolayer cells. In addition, our data demonstrated that aggressive properties of spheroid cells enriched with cancer stem cells contributes to ovarian cancer pathogenesis.

Contrary to previous reports that spheroid cells are more resistant to chemotherapy drugs [5]–[8], we demonstrated that spheroid cells were initially more sensitive than parent cells to cisplatin and paclitaxel treatment (**Fig. 6** and data not shown). However, our clonogenic survival assay indicated that more spheroid cells survived after 24 hrs of cisplatin treatment, compared with parent cells. This may be because spheroid cells proliferate faster than parent cells in CM, and therefore more dividing spheroid cells were killed by drug than their parent cells. However, after drug removal, the surviving spheroid cells recovered faster than parent cells. These two lines of evidence suggested that a large fraction of spheroid cells killed by cisplatin are more differentiated non-stem cells, while the small fraction of spheroid cells that survived chemotherapy are cancer stem cells. The stemness properties of these surviving spheroid cells was demonstrated by their ability to generate more tumor colonies than the large numbers of parent cells that survived from cisplatin treatment. These results may explain why current chemotherapeutic regimens are unable to achieve sustained remission in ovarian cancer patients, despite frequently producing a complete response at the time of first line treatment.

It has been shown that cancer cells use more glucose compared to normal cells and accumulate extracellular lactate even in normoxic conditions, a feature called Warburg effect [51], [52]. In addition, recent reports have shown that human pluripotent stem cells rely mostly on glycolysis to meet their energy demands [53], [54]. Using stable ^13^C-glucose isotope, we demonstrated that spheroid cells have a significant increase in glycolysis flux compared to their parent cells (**Fig. 7**). This pattern of glycolytic flux is similar to what has been observed for pluripotent stem cells [53]. The very different patterns of substrate utilization likely explain their aggressiveness and resistance to chemotherapies. Our results indicate that spheroid cells mainly route glucose to anaerobic glycolysis. This direct oxidation of glucose through anaerobic glycolysis is in accord with recent publications suggesting that CSCs may have even more active glycolytic activity compared to the bulk of general cancer cells [55]–[58]. In addition, we demonstrated that the amount of glucose utilized in the pentose phosphate pathway is far greater than that oxidized through the TCA cycle by spheroid cells. Since the oxidative branch of the pentose cycle is an efficient means for producing cytoplasmic NADPH and high NADPH production would be necessary for increased fatty acid synthesis, a NADPH dependent process, our data shows that synthesis of fatty acids are indeed increased in spheroid cells. Since hypoxia is associated with the formation and maintenance of cancer stem cells [59], our metabolic data suggest that ovarian CSCs are likely to survive in severely hypoxic microenvironments. These spheroid cell metabolic attributes may confer the increased chemoresistance observed in this study. On the contrary, parent cells display increased use of anabolic pathways to the detriment of anaerobic glycolysis and are likely to be more vulnerable to chemotherapy.
